# Evolution of Head and Neck Cutaneous Squamous Cell Carcinoma Nodal Staging—An Australian Perspective

**DOI:** 10.3390/cancers14205101

**Published:** 2022-10-18

**Authors:** Michael J. L. Hurrell, Tsu-Hui (Hubert) Low, Ardalan Ebrahimi, Michael Veness, Bruce Ashford, Sandro Porceddu, Jonathan R. Clark

**Affiliations:** 1 Department of Head and Neck Surgery, Chris O’Brien Lifehouse, Sydney, NSW 2050, Australia; 2 Department of Otolaryngology—Head & Neck Surgery, Faculty of Medicine and Health Sciences, Macquarie University, Sydney, NSW 2109, Australia; 3 Sydney Medical School, Faculty of Medicine and Health Sciences, University of Sydney, Sydney, NSW 2006, Australia; 4 Medical School, College of Health and Medicine, Australian National University, Canberra, ACT 2601, Australia; 5 Westmead Hospital, University of Sydney, Westmead, NSW 2006, Australia; 6 School of Medicine, University of Wollongong, Wollongong, NSW 2522, Australia; 7 Illawarra Health and Medical Research Institute, Wollongong, NSW 2500, Australia; 8 Illawarra Shoalhaven Local Health District, Wollongong, NSW 2502, Australia; 9 Radiation Oncology, University of Queensland, St Lucia, QLD 4072, Australia; 10 Princess Alexandra Hospital, Brisbane, QLD 4102, Australia; 11 Royal Prince Alfred Institute of Academic Surgery, Sydney Local Health District, Sydney, NSW 2050, Australia

**Keywords:** squamous cell carcinoma, head and neck cancer, lymphatic metastasis, cancer staging, prognosis, cutaneous, skin

## Abstract

**Simple Summary:**

Australia has the highest incidence of cutaneous squamous cell carcinoma of the head and neck (HNcSCC) in the world. Although the majority of HNcSCCs are cured by simple surgical excision, those that spread to lymph nodes require aggressive and debilitating surgery in conjunction with radiation therapy, with a significant risk of treatment failure and subsequent loss of life. Cancer staging is critical to guide prognosis, treatment (to maximise disease control and minimise morbidity), and for research. Australian institutions have been at the forefront of prognostication for HNcSCC with lymph node spread. Despite this, the search for a well performing staging system is ongoing. This review chronologically explores and summarises the Australian contribution to date and highlights the ongoing challenges.

**Abstract:**

Cutaneous squamous cell carcinoma of the head and neck (HNcSCC) is one of the commonest malignancies. When patients present with regional metastatic disease, treatment escalation results in considerable morbidity and survival is markedly reduced. Owing to the high incidence, Australian institutions have been at the forefront of advocating for reliable, accurate, and clinically useful staging systems that recognise the distinct biological characteristics of HNcSCC. As a result, an extensive body of literature has been produced over the past two decades, which has defined critical prognostic factors, critiqued existing staging systems, and proposed alternative staging models. Notwithstanding, a suitable staging system has proved elusive. The goal of cancer staging is to group patients according to cancer characteristics for which *survival* differs between groups (distinctiveness), consistently decreases with increasing stage (monotonicity), and is similar within a group (homogeneity). Despite implementing major changes based on published data, the latest edition of the American Joint Committee on Cancer (AJCC) staging manual fails to satisfy these fundamental requirements. This review chronologically explores and summarises the Australian contribution to prognostication and nodal staging of HNcSCC and highlights the ongoing challenges.

## 1. Introduction

Cutaneous squamous cell carcinoma (cSCC) is one of the commonest malignancies, with most cases presenting on the sun-exposed head and neck region [1,2,3,4]. Australia is recognised as having the highest incidence in the world with annual rates exceeding 2875 per 100,000 men over the age of 60 years living in coastal New South Wales [5]. Even though regional metastases occur in <5% of patients with head and neck cSCC (HNcSCC), they are associated with a marked reduction in survival despite aggressive multimodal therapy [1,2,3,4,6,7]. Morbidity also increases with nodal involvement requiring regional dissection and post-operative radiotherapy (PORT), which is offered to the majority of patients.

Unfortunately, cancer registry data on primary HNcSCC are lacking as non-melanoma skin cancer (NMSC) is not a notifiable cancer in most countries [8]. Distinguishing between disease-specific and all-cause mortality in a cancer that predominantly affects the elderly population is impossible without high-quality registry data. This lack of data has caused researchers to rely on single and multi-institutional data with inherent referral and treatment biases to predict survival statistics. Due to the frequency of HNcSCC, Australian institutions have been at the forefront of advocating for reliable, accurate, and clinically useful staging systems that recognise the distinct biological characteristics of HNcSCC from other SCC sites. As a result, an extensive body of literature defining critical prognostic factors in advanced HNcSCC has been produced by Australian cancer centres. Despite this, a suitable staging system has proved elusive notwithstanding multiple iterations of the American Joint Committee on Cancer (AJCC) staging manual since the first edition was introduced in 1978 (AJCC 1st edition).

A clinically useful cancer staging system should provide accurate prognostic information that facilitates appropriate management decisions, including treatment escalation and de-escalation where appropriate [9]. More specifically, the goal of cancer staging is to group patients according to cancer characteristics for which *survival* differs between groups (distinctiveness), consistently decreases with increasing stage (monotonicity), and is similar within a group (homogeneity) [10,11,12,13]. Despite implementing major changes based on published data, the eighth edition of the AJCC staging manual (AJCC 8th edition) fails to satisfy these fundamental requirements when interrogated using data from multiple institutions. The following sections explore the development of the knowledge that has shaped our approach to metastatic HNcSCC and aims to highlight the challenges and ongoing shortcomings of nodal staging.

## 2. Staging Systems

### 2.1. Parotid vs. Cervical Nodes (P/N)

The AJCC 1st edition identified the parotid and cervical nodes as important nodal basins for NMSC cancers of the head and neck [14]. In clinical practice, the parotid is the most frequent site of HNcSCC to develop nodal metastasis [3,6,15]. In Australia, the commonest parotid malignancy overall is metastatic HNcSCC and not a primary parotid malignancy [6,8,16]. In 2001 O’Brien investigated whether treatment of the clinically negative neck was warranted in patients with parotid metastasis (cP + N0) and found that 35% of patients with metastatic cSCC in the parotid had occult neck disease [6]. The following year, O’Brien devised a nodal staging system for metastatic HNcSCC which differentiated between parotid and neck disease reporting that more advanced parotid (P) disease reduced regional (parotid) control while more advanced cervical node (N) disease reduced survival [3]. This staging system, known as the P/N system, is shown in Table 1.

**Table 1:** Comparison of the P/N, ITEM, N1S3, AJCC7, and AJCC8 staging systems in relation to high-risk HNcSCC.

	P/N [3]	ITEM [17]	N1S3 [18]	AJCC7 [19] (“Cutaneous SCC and Other Cutaneous Carcinomas”)	AJCC8 [20] (“Cutaneous SCC of the Head and Neck”)
**Primary tumour categories**	Nil	Margin status: involved/clear (1.0/0)	Nil	TX: primary tumour cannot be assessed	TX: primary tumour cannot be identified
				T0: no evidence of primary tumour	Tis: carcinoma in situ
				Tis: carcinoma in situ	T1: tumour < 2 cm
				T1: tumour 0–2 cm with less than two high-risk features *	T2: tumour 2–<4 cm
				T2: tumour > 2 cm or tumour any size with more than two high-risk features *	T3: tumour ≥4 cm or minor bone erosion or PNI ^ or deep invasion #
				T3: tumour with invasion of maxilla, mandible, orbit, or temporal bone	T4a: tumour with gross cortical bone/marrow invasion
				T4: tumour with invasion of skeleton (axial or appendicular) or PNI of skull base	T4b: tumour with skull base invasion and/or skull base foramen involvement
**Regional metastasis categories**	P1: Metastatic parotid node 0–3 cm	ENE: yes/no (4.8/0)	I: single lymph node 0–3 cm	NX: regional lymph nodes cannot be assessed	NX: regional lymph nodes cannot be assessed
	P2: Metastatic parotid node >3–6 cm or multiple parotid nodes		II: single lymph node >3 cm or multiple lymph nodes 0–3 cm	N0: no regional lymph node metastases	N0: no regional lymph node metastases
	P3: metastatic parotid node >6 cm or disease involving facial nerve or skull base		III: multiple lymph nodes >3 cm	N1: metastasis in a single impsilateral node, 0–3 cm	N1: metastasis in a single impsilateral node, 0–3 cm and ENE(−)
	N0: no clinical neck disease			N2a: metastasis in a single ipsilateral lymph node, >3–6 cm	N2a: metastasis in a single ipsilateral or contralateral node 0–3 cm and ENE(+) OR single ipsilateral node >3–6 cm and ENE(−)
	N1: single ipsilateral neck node 0–3 cm			N2b: metastasis in multiple ipsilateral lymph nodes, none >6 cm	N2b: metastasis in multiple ipsilateral nodes, non >6 cm and ENE(−)
	N2: single node >3 cm or multiple neck nodes or contralateral neck nodes			N2c: metastasis in bilateral or contralateral lymph nodes, none >6 cm	N2c: metastasis in bilateral or contralateral lymph nodes, none >6 cm and ENE(−)
				N3: metastasis in a lymph node, >6 cm	N3a: metastasis in a lymph node, >6 cm and ENE(−)
					N3b: metastasis in a single ipsilateral lymph node >3 cm and ENE(+) or multiple ipsilateral, contralateral, or bilateral nodes, any with ENE(+)
**Distant metastasis categories**	Nil	Nil	Nil	M0: no distant metastases	M0: no distant metastases
				M1: distant metastases	M1: distant metastases
**Other categories**	Nil	Immunosuppression: yes/no (1.8/0)	Nil	Nil	Nil
		Treatment: surgery + PORT/surgery only (−1.8/0)			
**Overall Prognostic Stages:**	P1N0-N2	Low risk: overall score < 2.6	I	Stage 0: TisN0M0	Stage 0: TisN0M0
	P2N0-2	Moderate risk: overal score >2.6–3	II	Stage I: T1N0M0	Stage I: T1N0M0
	P3N0-2	High risk: overall score >3	III	Stage II: T2N0M0	Stage II: T2N0M0
				Stage III: T3N0M0 or T1-3N1M0	Stage III: T3N0M0 or T1-3N1M0
				Stage IV: T1-3N2M0 or TanyN3M0 or T4NanyM0 or TanyNanyM1	Stage IV: T1-3N2M0 or T(any)N3M0 or T4N(any)M0 or T(any)N(any)M1

* High-risk features for AJCC7 primary tumour categories T1 and T2: (1) >2 mm thickness; (2) Clark level ≥ IV; (3) PNI; (4) Primary site ear; (5) Primary site hair-bearing lip; (6) Poorly differentiated or undifferentiated; ^ PNI for AJCC8 primary tumour category T3: Tumour cells within the nerve sheath of a nerve lying deeper than the dermis or measuring 0.1 mm or larger in calibre, or presenting with clinical or radiographic involvement or named nerves without skull base invasion or transgression; # Deep invasion for AJCC8 primary tumour category T3: Invasion beyond subcutaneous fat or >6 mm.

In 2006, the P/N system was tested in an international cohort of 322 HNcSCC patients with regional metastases. Disease specific survival (DSS) was significantly worse with advanced clinical P stage and where both the parotid and neck nodes were involved pathologically (P+N+) [16]. Whilst the rationale for separating the P and N stage to guide treatment was sound, the prognostic utility was less clear. However, it highlighted deficiencies within the AJCC staging system of the time [21] and encouraged further research into regional disease behaviour and staging.

### 2.2. ITEM (Immunosuppression, Treatment, Extracapsular Spread, and Margins) Score

In 2009, Veness and colleagues developed a novel risk stratification system based on a series of 250 HNcSCC patients with regional metastasis [17]. Multiple prognostic variables were evaluated including immunosuppression, location, treatment, lymph node size, lymph node number, O’Brien P/N stage, extranodal extension (ENE) previously termed extracapsular spread (ECS), and margin status. Four variables were found to predict DSS on multivariate analysis: immunosuppression, treatment, ENE, and margin status (ITEM). A risk score was obtained based on the regression coefficients and cut-off points were used to define low-, moderate-, and high-risk groups (see Table 1). When applied to the study population, the 5-year risk of death from HNcSCC for patients with high-risk (>3.0), moderate-risk (>2.6–3.0), and low-risk (2.6) ITEM scores was 56%, 24%, and 6%, respectively. Unfortunately, since the score includes treatment and histopathological variables, it cannot be used at presentation to guide treatment escalation or de-escalation.

### 2.3. N1S3

The following year (2009), the N1S3 system was published [18]. Forest et al. performed a multivariate analysis on 215 HNcSCC patients with regional metastasis. On univariate analysis the nodal number, size, and ENE predicted survival. For simplicity, cut-offs for nodal number and size were aligned with the AJCC staging system for mucosal head and neck cancer. Nodes from the parotid and neck were incorporated together and the system classified patients into three stages (see Table 1). The system was externally validated in another Australian cohort of 250 HNcSCC patients with regional metastases; N1S3 discriminated well between the three stages for regional control and DSS. Whilst N1S3 is a simple but efficacious system, the authors commented that “other factors such as ENE, immunosuppression and treatment modality were equally important but less readily applied” [18].

## 3. AJCC 7th Edition

The seventh edition of the AJCC staging manual (AJCC 7th edition), released in 2010, was the first to stratify patients based on nodal number, size, and laterality (Table 1) [19]. The new N category and overall staging criteria was a replica of the mucosal staging from the same edition. Whilst a unified system promotes acceptance through familiarity [22], it ignores the distinct biological differences between SCC of cutaneous and mucosal origin. This meant that T1N2M0 disease was grouped together with T4N3M1 disease as stage IV. In 2011, Brunner et al. evaluated the appropriateness of including N2 and N3 disease into stage IV in a multi-institutional cohort of 603 HNcSCC patients with regional metastasis [23]. They found that patients with distant disease (M1) had a very poor, and markedly different prognosis to those with only regional disease, with five-year DSS for N2, N3, and M1 of 75%, 65%, and 11%, respectively. In addition, the 35 M1 patients from the study were all initially treated with curative intent but developed distant metastasis during follow-up suggesting that the true impact of distant metastases was likely to be worse and it was therefore recommended that regional disease be grouped independently from distant metastases regardless of extent.

### 3.1. AJCC 7th Edition vs. N1S3

In 2012, Clark et al. compared N1S3 directly with the new AJCC 7th edition nodal staging system in an Australian multi-centre cohort (Table 2) [13]. Cox regression was used to adjust for treating institutional as well as clinicopathological variables such as immunosuppression, PORT, nodal margins, and ENE. The authors suggested that the complexity of the AJCC 7th edition was unhelpful as there was no prognostic distinctiveness for any N2 subgroupings, in particular N2c, which was rarely designated. The proportion of variation explained (PVE) was similar for both staging models indicating that the performance of the two models was similar, but N1S3 distributed patients better and stratified patients better according to risk of death.

### 3.2. AJCC 7th Edition Cutaneous vs. Oral

In 2014, Brunner et al. explored the justification for replicating the mucosal SCC N category in the AJCC 7th edition for HNcSCC, noting the different disease behaviour and patient populations [1]. The study consisted of 672 patients with regional metastatic HNcSCC and 225 patients with regional metastatic oral cavity SCC treated with curative intent. The oral SCC N category stratified patients well according to survival, whereas HNcSCC stratification was poor. Within the HNcSCC N2 subgroups there was only 1% separation of DSS at 3 years. Adjusting for immunosuppression, age, gender, PORT, treatment institution, and ENE, the increase in risk by N category was neither clinically useful nor monotonic. In summary, the AJCC 7th edition worked well for mucosal SCC but performed poorly for HNcSCC, providing evidence for a standalone staging system for HNcSCC regional disease.

## 4. AJCC 8th Edition

HNcSCC was recognised as a separate entity for the first time in the AJCC 8th edition, published in 2017 [20]. Significant changes were made to the T category, but the N category remained aligned with mucosal SCC, with the introduction of ENE for the first time.

### 4.1. AJCC8 vs. AJCC7

In a single institution analysis of 382 patients, Liu et al. compared the performance of the AJCC 7th edition versus the AJCC 8th edition [9]. In this cohort, 74.6% of patients were upstaged by the AJCC 8th edition due to the addition of ENE. Although risk stratification by N category was poor for the AJCC 7th edition, the results for the AJCC 8th edition were inferior, with no risk stratification observed between any nodal subgroups for either DSS or OSS. When translated to stage, 27.7% of patients were upstaged by the AJCC 8th edition, with 88% of patients with regional metastases designated stage IV versus 60% for AJCC 7th edition. Stage III and IV Kaplan–Meier curves overlapped for both DSS and OS. Similar results were reported in another study by Sood and colleagues [24].

In 2020, Luk et al. evaluated the AJCC 8th edition and compared its prognostic utility in an Australian multicentre study of 1146 HNcSCC patients with regional metastasis [25]. In this cohort, the N category was upstaged in 80.9% of patients by the AJCC 8th edition due to the presence of ENE, 29.9% were upstaged from stage III to IV, resulting in 90.6% of patients with nodal metastases being assigned to stage IV disease. The prognostic performance of the AJCC 8th edition N category was poor according to Harrell’s concordance index (C-index) (0.62 for DSS and 0.59 for OS) with no risk stratification observed between pN1, pN2a, pN2b, or pN2c for DSS. With regards to stage, the AJCC 8th edition performance was poor with C-indexes of 0.54 for DSS and 0.53 for OS; almost equivalent to “flipping a coin”. This study also showed that the N3a nodal category (metastatic node >6 cm without ENE) is likely redundant, being reported in only 0.3% of patients and N3a disease carrying a worse prognosis than N3b.

### 4.2. AJCC 8th Edition vs. N1S3 vs. ITEM

Ebrahimi et al. compared the prognostic performance of the AJCC 8th edition against the N1S3 and ITEM systems in a multicentre cohort of 990 patients [26]. Model performance was measured using PVE, C-index, Akaike Information Criterion (AIC), and Bayesian Information Criterion (BIC). The AJCC 8th edition N category distribution was poor with 84% of patients classified as either N2a or N3b due to the inclusion of ENE, whereas N2c and N3a appeared redundant. For the AJCC 8th edition, the majority of patients (90.6%) were grouped as stage IV. In comparison N1S3 and ITEM both exhibited a reasonably balanced distribution (N1S3 stage I, II, and III was 39.3%, 44.9%, and 15.8%, respectively; ITEM low risk, moderate risk, and high risk was 18.1%, 35.5%, and 46.5%, respectively). However, only 34.3% of patients were allocated to the same risk level by both N1S3 and ITEM reflecting the different variables used in each system.

For DSS, N1S3 performed similarly to the AJCC 8th edition N category and the Kaplan–Meier curves for N1S3 appeared to stratify patients well. Although N1S3 was preferred over the AJCC 8th edition due to simplicity, the C-index of 0.62 and PVE of 10.9% was relatively poor compared with other cancer staging models. A further finding was that all staging systems were better predictors of DSS than OS. This reflects the competing risk of age-related comorbidities in the elderly HNcSCC population, with a median age of 73 years and relatively high disease control rates. They recommended the use of DSS as the preferred endpoint for HNcSCC nodal staging rather than OS.

### 4.3. AJCC 8th Edition Within-Stage Heterogeneity

In view of the poor performance of the AJCC 8th edition and the fact that most patients appear to be classified as pN2a, pN3b, and stage IV, Ebrahimi and colleagues hypothesized that patients with widely different prognoses were being classified into the same risk group. To identify sources of within-stage prognostic heterogeneity, a cohort of 1146 HNcSCC patients with regional metastases was analysed [27]. Conclusions regarding within-group homogeneity were not possible for pN2c or pN3a groups due to insufficient numbers. However, pN2a and pN3b exhibited significant heterogeneity due to the effect of immunosuppression, nodal size, nodal number, and perineural invasion (PNI). Within pN2a, patients without PNI or immunosuppression had a 5-year DSS of 87.1%, compared with 69.9% in the presence of either feature. Regarding stage, TNM stage III contained only pN1 patients, and was relatively homogenous; however, stage IV contained over 90% of the entire cohort with marked heterogeneity observed. Ebrahimi et al. created low, moderate, and high-risk subgroups in an exploratory analysis of the stage IV patients, based on immunosuppression, PNI, ENE, and the number and size of the nodal metastases, weighted according to the hazard ratios observed in the multivariate analysis. The 5-year DSS for the low-risk stage IV patients was 90%, whereas the 5-year DSS for the high-risk stage IV patients was only 60%. These results revealed a staggering 400% difference in disease-specific mortality risk for patients ultimately staged together by the AJCC 8th edition, largely due to the incorporation of ENE, with resulting within-stage heterogeneity contributing significantly to the overall poor performance.

## 5. Additional Research of Relevance to Nodal Staging

### 5.1. Low-Risk

Most studies have focused on identifying high-risk patients requiring treatment escalation. In 2011, Ebrahimi et al. published on their efforts to identify a subset of patients who could benefit from treatment de-escalation [28]. Using the N1S3 system, 168 “low-risk” metastatic HNcSCC patients (with a single lymph node measuring ≤3 cm) who had undergone surgery as their primary treatment were analysed, of which 20% did not receive PORT. Reasons included patient refusal, poor performance status, physician preference based on favourable histopathological features, and a prior history of RT to the surgical bed. Amongst this cohort, the regional control rate was 92% and the remaining 8% who recurred were successfully salvaged with further surgery and PORT. The 5-year DSS was 100% for patients without ENE versus 93% for those with ENE, suggesting that N1S3 stage I patients without ENE or other adverse clinicopathological features could be treated with surgery alone. However, the authors emphasised the prerequisite for patients with regional disease to have undergone appropriate parotidectomy and neck dissection, based on the location of the primary tumour, before considering the omission of PORT.

### 5.2. Soft Tissue Metastases (STM)

The prognostic impact of STM has been demonstrated in melanoma and mucosal SCC of the HN [29,30]. In 2012, Kelder et al. compared the prognostic difference between STM and ENE in 164 HNcSCC patients with regional metastasis [31]. Adjusting for the effect of age, margin status, primary lesion size, number of nodes, and PORT, the presence of STM was associated with worse survival than ENE, suggesting that pathology reports for HNcSCC should include not just the presence of ENE, but also the presence, number, and size of STMs. Furthermore, a lower threshold for aggressive multimodal treatment in the presence of STM was recommended.

After adopting their own recommendations, the same group published an updated analysis on ENE and STM in 2020 on a series of 535 HNcSCC patients with regional metastasis [32]. Interestingly, only 22.4% had nodal disease without either ENE or STM versus 35.4% in 2012 and 26.2% had STM alone versus 13.4% in 2012. The finding of STM in the absence of ENE had doubled and STM in the presence of ENE had reduced, suggesting that the increased recognition of STM had led to both increased identification and the reclassification of cases previously labelled as ENE. Whilst ENE alone, STM alone, and ENE with STM were all associated with reduced OS, the impact of STM was now similar to that of ENE. The diminished survival impact of STM was considered a stage migration effect associated with greater recognition and more aggressive treatment of STM when identified compared with the results of the 2012 study. Based on this evidence it was recommended that STM be managed equivalent to ENE from a prognostic and treatment perspective, but that these findings should be individually documented in pathology reports to facilitate further study.

### 5.3. Disease-Free Interval (DFI)

Time to development of lymph node metastases in melanoma patients has been shown to be prognostic [33]. Consequently, Ebrahimi et al. in 2012 investigated whether the time interval between treatment of an index primary HNcSCC and subsequent presentation of regional metastatic disease impacted survival outcomes [34]. With a cohort of 229 HNcSCC patients with regional metastasis, only 20% of patients presented with concurrent primaries and regional metastatic disease. In the remaining patients, the mean and median DFI were 11.7 and 7.2 months, respectively. On multivariate analysis, DFI (≤9 vs. >9 months) was associated with locoregional control and DSS. The authors concluded that DFI was likely to be an inverse surrogate for aggressive disease biology, and unlike many other variables proposed for staging, DFI is available to clinicians before definitive treatment is delivered.

### 5.4. Lymph Node Ratio (LNR)

Several studies have suggested that the LNR may be a superior prognosticator compared to the number of involved nodes in HNcSCC [35,36,37]. In 2017, Vasan et al. examined the utility of LNR in a cohort of 326 HNcSCC patients with regional metastasis [38]. For both DFS and OS, a significant decrease in survival was found for patients with a LNR >6% with almost double the risk of death after adjusting for the effects of other covariates. Surprisingly, unlike oral SCC [39], nodal yield was not an independent predictor of survival. However, when 18 or more nodes were removed, Vasan et al. determined that a LNR >6% was a suitable threshold to stratify patients into low-risk and high-risk categories based on a 3-year OS difference of 17.5%.

### 5.5. Number of Nodal Metastases

The AJCC staging manual has only considered the nodal number as single versus multiple for HNcSCC. In 2019, Sood et al. found a cumulative increase in risk with each additional involved node [40]. In a large multicentre study involving 1128 HNcSCC patients with regional metastasis, Ebrahimi et al. confirmed that number of nodal metastases was an independent predictor of survival and provided additional prognostic information compared with AJCC 8th edition [41]. Model performance was compared against the AJCC 8th edition using C-index, PVE, AIC, and BIC. Exploratory analyses determined the optimal cut-offs to be 1–2 versus 3–4 versus ≥5 nodes with good distribution amongst these groups. Using this categorisation, the risk of disease-specific death was 1.6 times higher for the 3–4 group, and 2.9 times higher for the ≥5 group. Interestingly, the hazard ratios remained consistent despite adding the N category and overall stage in the multivariate model, suggesting that incorporating the number of nodes provides additional prognostic information to the AJCC 8th edition. When the prognostic performance of this simple categorical variable was compared to the AJCC 8th edition staging using objective measures and adjusting for institution and treatment, the categorical number of nodes was superior in to the AJCC 8th edition TNM stage and equivalent to the AJCC 8th edition N category, but with superior distribution and parsimony providing strong support for incorporating the nodal number into future HNcSCC staging systems.

### 5.6. Location—Parotid vs. Neck

In 2018, the results of the Trans-Tasman Radiation Oncology Group (TROG) randomized phase III trial were published, where the addition of concurrent chemotherapy (weekly carboplatinum) to PORT failed to improve locoregional control or survival in high-risk HNcSCC [42]. In this study, the presence of parotid metastasis was considered a high-risk criterion satisfying inclusion. Mooney et al. in 2021 aimed to re-evaluate the impact of the location of nodal metastasis on survival in patients with HNcSCC [43]. Of the 535 patients, 235 had parotid metastases in isolation, 96 had neck metastases alone, and 204 had both. On univariate analysis, disease in the neck resulted in worse DSS and OS compared to isolated parotid disease. On multivariate analysis, this finding persisted and further analysis demonstrated that patients with multiple parotid nodes behaved similarly to those with a single involved neck node and that patients with multiple cervical nodal deposits had the lowest survival.

### 5.7. A Hierarchical Approach to Nodal Staging

**Figure 1:**
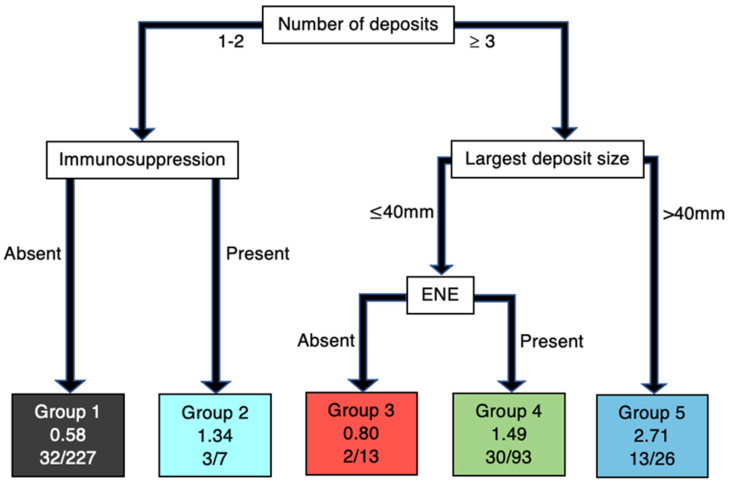
DSS Analysis. RPA decision tree. Groups 1–5 represented in leaves with Cox hazard ratio and DSS ratio (number of events observed in group/overall group number).

**Figure 2:**
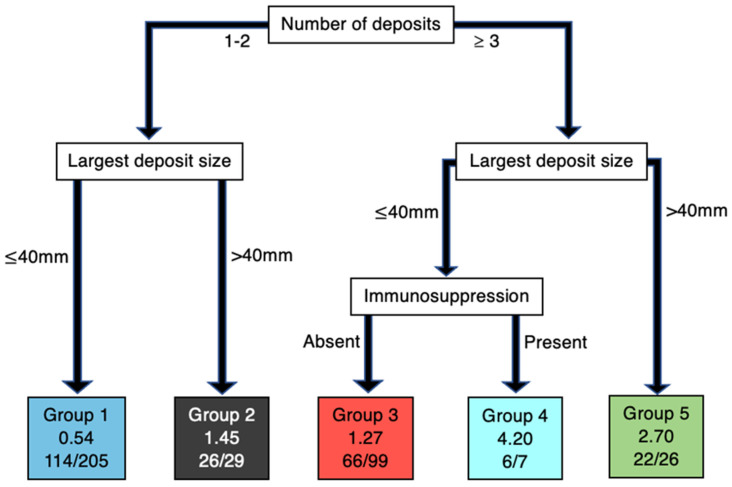
OS analysis. RPA decision tree. Groups 1–5 represented in leaves with Cox hazard ratio and OS ratio (number of events observed in group/overall group number).

Clinicopathologic characteristics considered in the AJCC 8th edition are proven prognosticators, but the hierarchy or order in which these variables should be considered is not well understood. Recently, Hurrell et al. utilised recursive partitioning analysis (RPA), a statistical method for prioritising variables and creating homogenous groups, to determine the impact and hierarchy of these variables on a cohort of 366 HNcSCC patients with regional metastasis [44]. The RPA for DSS revealed that the number of metastatic deposits (including STM) was of primary significance, followed by deposit size and the presence of immunosuppression together. ENE was significant at the third level of hierarchy (Figure 1). The RPA for OS also revealed that the number of deposits carried the highest order of significance. Deposit size was significant at the second level, followed by immunosuppression at the third level. ENE was not found to significantly influence OS. (Figure 2). Following the RPA, three clinically relevant N categories were created with the four key clinicopathological variables from the RPA, as follows:

1. Low risk:
  ≤2 Metastases and no immunosuppression
  or
  ≥3 Metastases, <40 mm and no ENE
2. Moderate risk:
  ≤2 Metastases and immunosuppression
  or
  ≥3 Metastases, <40 mm and ENE
3. High risk:
  ≥3 Metastases and >40 mm

**Figure 3:**
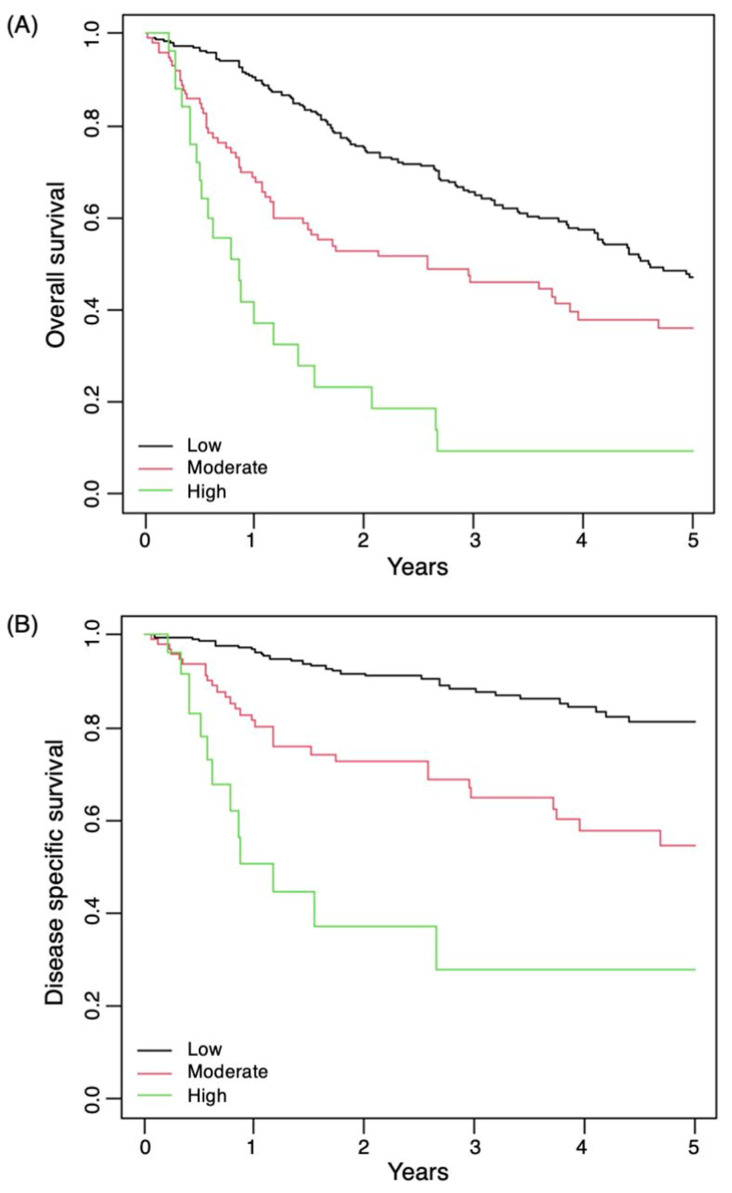
Kaplan–Meier curves with low, moderate, and high-risk groupings. (**A**) OS. (**B**) DSS.

Using these three risk categories, it was found that 66% of patients were low-risk, 27% of patients were moderate-risk, and 7% of patients were high-risk, with good separation on Kaplan–Meier curves (Figure 3).

## 6. Discussion

This article has summarised the Australian contribution to prognostication and staging of regionally metastatic HNcSCC. Prior to the AJCC 7th edition, all regional disease was designated to a single group (N1), implying that the mere presence or absence of disease in the nodal basins was the only relevant prognosticator [10]. The AJCC 7th edition was the first edition to recognise the importance of the extent of regional disease by adopting the same nodal staging used for mucosal HN SCC based on the size, number, and laterality of involved lymph nodes [19]. The AJCC 8th edition recognised HNcSCC as a unique entity but replicated the nodal staging used for oral mucosal SCC where at the same time, distinct staging systems were introduced for other biologically disparate HN cancers such as human papilloma virus (HPV)-related oropharyngeal SCC.

HNcSCC is biologically distinct from mucosal HN SCC, with different aetiological factors, demographics, and genetics. Metastatic HNcSCC is the most highly mutated human cancer due to life-long exposure to ultraviolet (UV) radiation, with tumour mutation burdens several folds higher than HN mucosal SCC [45,46,47,48]. Furthermore, regional metastases from HNcSCC are usually metachronous from the primary tumour and in many cases (20–30%), the primary or index lesion cannot be identified. This makes applying the standard approach for combining T and N categories problematic and in practice, this rarely happens. Finally, the cumulative UV exposure that induces HNcSCC means that most patients affected are elderly, with approximately 32% of patients developing nodal metastases being over the age of 80 years and many with multiple comorbidities [49]. Thus, applying OS, the standard staging endpoint, is problematic because it does not adequately reflect the impact of HNcSCC. While statistical methods, such as multivariable models adjusting for age and competing-risk techniques can help overcome this, they still rely on accurate cause of death determination. This is difficult to obtain without cancer registry data, particularly in geographically sparse countries such as Australia where distance creates barriers to patients returning for follow up.

Developing simple, accurate, and clinically relevant staging systems is challenging. Staging is not amenable to being tested or validated in prospective studies. Complex staging systems and nomograms are difficult to apply in routine clinical practice. They also tend to overfit data from which they were derived and then fail to perform in the real world. This problem highlights the need for proposed systems to be validated in multiple external cohorts before being introduced into clinical practice. In contrast, simple staging systems inappropriately combine heterogeneous groups together. Disappointingly, the AJCC 7th edition and AJCC 8th edition have failed on multiple fronts by adopting complex staging criteria with marked intra-stage heterogeneity. For example, in the AJCC 8th edition, the introduction of ENE to the pathological N category resulted in approximately 80% of patients being upstaged to stage IV, incorrectly suggesting a poor prognosis in what is a highly curable disease. Cancer staging is a dynamic process and must evolve over time as treatment and prognosis change. Hasmat et al. showed that the risk of HNcSCC-related mortality in 2010–2017 was approximately one third (HR = 0.30, *p* = 0.001) of that in 1980–1989 reflecting more consistent and improved multidisciplinary care, particularly in elderly patients who may have been previously treated conservatively [49]. This is likely to improve further with the introduction of immune check point inhibitors (ICI) which are currently redefining what was once incurable disease. ICIs clearly highlight how a staging system can rapidly become obsolete when new therapeutic modalities arise.

The optimal metrics for assessing cancer staging are contentious. The wide array of statistical techniques utilised to analyse data can be bewildering and understanding their role is important when critiquing published data. The concepts of distinctiveness, monotonicity, and homogeneity are intuitive to clinicians wanting to group patients according to prognosis but are hard to quantify. Clinicians have become familiar with Kaplan–Meier survival curves; however, survival curves do not provide information about within-group heterogeneity and without confidence intervals and robust sensitivity analyses the precision and reliability of groupings is unknown. Many studies will use statistical tests that are applicable to time-to-event analyses and conclude that a staging system is valid because the log-rank test or Cox proportional hazards model yielded a significant p value, without considering the fundamental role of staging, i.e., to inform prognosis. In contrast, metrics such as PVE, AIC, BIC, and C index are abstract measures of model performance that are challenging to understand. PVE is a measure of how much of the variability in outcome (e.g., death) can be “explained by” variation in the other (e.g., ENE) variables. It is possible that the C index is the intuitive performance metric to apply; this is a goodness of fit measure for models which produce risk scores where a C index of 0.5 represents a random binary event (e.g., flipping a coin) and a C index of 1.0 represents perfect prediction. In contrast, both AIC and BIC yield numbers are meaningless in isolation and need to be compared with other models. Whilst these performance metrics quantify how well a model predicts the outcome of interest, e.g., death from any cause (OS) or death from cancer (DSS), they do not provide information on how distinct groups are from each other or whether they provide a monotonic increase in risk. In summary, while a single staging metric is appealing, multifaceted analyses of performance are required to understand whether a proposed staging system is truly superior. Thus far, there has been no published staging system that performs ‘well’, with C indices typically around 0.6.

To develop more informative staging systems, alternative approaches are required that incorporate biological characteristics and novel statistical approaches; RPA is an alternative approach to traditional proportional hazards modelling which combines patients into groups based on the hierarchy of importance of prognostic variables. RPA gained significant attention in the HN cancer literature when applied to oropharyngeal cancer by Ang et al. [50], who stratified patients according to HPV status and tobacco use. In a similar way, immune competency and molecular profiling may enable more predictive staging models for HNcSCC but remains to be proven. Any new approach needs to be validated in multiple cohorts before advocating for change. In the long term, machine-based learning approaches coupled with cancer registry data may be the optimal approach to accurate prognostication that can evolve along with new treatments and improvements in cancer care.

## 7. Conclusions

Developing a clinically relevant and accurate staging system for HNcSCC has proven elusive. The lack of reliable cancer registry data has contributed to this challenge. Australian institutions have contributed extensively to defining prognostic criteria in HNcSCC, critiquing the existing staging systems, and proposing alternative models. HNcSCC is a distinct biological entity that warrants a staging system distinct from mucosal HN SCC that considers its carcinogenesis and demographics in addition to the extent of disease.

## Figures and Tables

**Table 2:** Comparison of the AJCC7 and N1S3 staging systems for HNcSCC nodal disease.

AJCC7 N Stage				N1S3			
Stage	Patient Distribution (%)	5-Year DSS (%)	HR *	Stage	Patient Distribution (%)	5-Year DSS (%)	HR *
N1	42.1	83	-	I	42.5	83	-
N2a	9.6	79	1.1	II	43.1	78	1.4
N2b	40.1	74	1.5	III	14.4	63	2.1
N2c	2	81	1.4				
3	6.1	65	2.1				

* HR = Hazard Ratio.

## Data Availability

No data reported (except what is referenced in the text).
